# Experimental comparison of two methods to study barley responses to partial submergence

**DOI:** 10.1186/s13007-021-00742-5

**Published:** 2021-04-13

**Authors:** Alexandra Miricescu, Tomás Byrne, Catherine M. Doorly, Carl K. Y. Ng, Susanne Barth, Emmanuelle Graciet

**Affiliations:** 1 Department of Biology, Maynooth University, Maynooth, Kildare Ireland; 2 Kathleen Lonsdale Institute for Human Health Research, Maynooth University, Maynooth, Kildare Ireland; 3 Crop Science Department, Teagasc Crops, Environment and Land Use Program, Oak Park, Carlow R93XE12 Ireland; 4 School of Biology and Environmental Science, Centre for Plant Science, UCD Earth Institute, O’Brien Centre for Science West, University College Dublin, Belfield, Dublin, D04 N2E5 Ireland

**Keywords:** Barley, Waterlogging, Screening, Crop improvement

## Abstract

**Background:**

Crop yield is dependent on climate conditions, which are becoming both more variable and extreme in some areas of the world as a consequence of global climate change. Increased precipitation and flooding events are the cause of important yield losses due to waterlogging or (partial) submergence of crops in the field. Our ability to screen efficiently and quickly for varieties that have increased tolerance to waterlogging or (partial) submergence is important. Barley, a staple crop worldwide, is particularly sensitive to waterlogging. Screening for waterlogging tolerant barley varieties has been ongoing for many years, but methods used to screen vary greatly, from the type of soil used to the time at which the treatment is applied. This variation makes it difficult to cross-compare results.

**Results:**

Here, we have devised a scoring system to assess barley tolerance to waterlogging and compare two different methods when partial submergence is applied with either water or a starch solution at an early developmental stage, which is particularly sensitive to waterlogging or partial submergence. The use of a starch solution has been previously shown to result in more reducing soil conditions and has been used to screen for waterlogging tolerance.

**Conclusions:**

Our results show that the two methods provide similar results to qualitatively rank varieties as tolerant or sensitive, while also affecting plants differently, in that application of a starch solution results in stronger and earlier symptoms than applying partial submergence with water.

**Supplementary Information:**

The online version contains supplementary material available at 10.1186/s13007-021-00742-5.

## Introduction

Plants in the wild continuously respond to a wide range of environmental cues, as well as stresses. Understanding how plants sense and mount a response to biotic or abiotic stresses is key to identifying genes that can be targeted for crop improvement. Screening and identifying germplasm or varieties that have increased tolerance to a particular stress is also crucial for future breeding strategies. Global climate change is likely to impact negatively on crop yields in many regions of the world, thus exacerbating the need to screen efficiently for varieties that exhibit increased tolerance to environmental stresses. For example, as a consequence of global climate change, several areas worldwide are predicted to have increased precipitation and flooding events, which have the potential to decrease crop production [1, 2]. Increased precipitation and/or poor soil drainage results in waterlogging (i.e. when roots only are in contact with the excess of water in the soil [3]) or (partial) submergence (when roots and parts of or all of the shoot are underwater [3]). In the last decade, waterlogging and submergence have been among the primary stresses that affected crop yield, highlighting the need to identify and generate germplasm that is more tolerant to this abiotic stress [1].

Plant survival and crop yield losses under waterlogged conditions depend on the duration and severity of the stress (i.e. whether the plant is completely or partially submerged) [4, 5], as well as the season and developmental stage at which waterlogging or (partial) submergence occurs [6–8]. For example, barley and wheat appear to be most sensitive to waterlogging immediately after germination (e.g. 2 weeks after sowing) and during flowering [7, 8], despite some variation depending on the studies and experimental conditions used [9]. Another important factor that affects plant tolerance to (partial) submergence is temperature [10, 11]. Importantly, the characteristics of the soil also play an important role for plant adaptation and response to waterlogging and (partial) submergence [7]. Soil composition may aggravate the effects of waterlogging by affecting the level of certain nutrients such as nitrates (whose availability may decrease), but also metals (e.g. manganese and iron levels may increase) [7]. Furthermore, waterlogging and partial submergence may change soil microbiome composition, resulting in the release of toxins [12, 13].

The main negative effect of waterlogging and (partial) submergence on plants is the reduced availability of oxygen (O_2_) due to reduced gas diffusion in water compared to air [14]. Importantly, both inward (from the atmosphere into the cells) and outward (from the cells towards the outside) gas diffusion is affected. For example, during waterlogging or (partial) submergence, the reduced inward diffusion of O_2_ results in lower intracellular O_2_ levels, which affects cellular respiration. Similarly, CO_2_ diffusion is also reduced, which affects photosynthesis [5]. In contrast, the decreased outward diffusion of gaseous phytohormones such as ethylene results in its intracellular accumulation. This serves as an essential signal to trigger the onset of plant responses to waterlogging and (partial) submergence [15, 16]. In the case of (partial) submergence, water turbidity also results in reduced light, which negatively affects photosynthesis [4, 5]. Decreased photosynthesis and respiration in turn negatively affect energy and carbohydrate levels. As a result, ATP production is based mainly on glycolysis. Other pathways such as ethanol fermentation play an important role to regenerate nicotinamide adenine dinucleotide [2, 17–20]. Waterlogging and (partial) submergence are typically followed by another stressful event after the water recedes and O_2_ levels increase again resulting in reoxygenation and an oxidative burst [15].

Barley is one of the most important cereal crops cultivated worldwide [21], but it is also particularly sensitive to waterlogging or (partial) submergence [7, 8], so that future yields could be threatened by the predicted increased precipitations and flooding events in the context of global climate change. In recent years, considerable efforts have been made to identify (i) waterlogging tolerant varieties [22–24], (ii) target genes that could be modified to increase waterlogging tolerance [25–29], and (iii) molecular markers for waterlogging tolerance [30]. Despite these efforts and the need to identify or generate germplasm in barley with increased tolerance to waterlogging, one major limitation has been the disparity of protocols used to assess tolerance/sensitivity to waterlogging or (partial) submergence. For example, while crucial, field experiments are inherently variable, as climatic conditions (e.g. temperature) and soil will unavoidably vary from year to year or in different geographical locations. In addition, in the field, plants may be subjected to multiple stresses that are not under control. Working in controlled conditions overcomes some of these problems, but protocols used and scoring schemes vary considerably. For instance, some protocols use natural soils [26], which makes it difficult to repeat the experiments, and applying waterlogging stress at different developmental stages may affect the outcome of experiments [9].

Here, we compared two protocols under controlled conditions to test barley responses to partial submergence when the stress was applied at an early developmental stage, when seedlings have 1–2 leaves (L1/L2 stage), thus mimicking partial submergence conditions in the field in autumn. We focused on winter barley varieties because they are expected to be particularly affected by climate change in the future due to their sowing time in the Northern hemisphere and their need to grow during the winter season. Based on a preliminary waterlogging field experiment conducted in Oak Park (Ireland [31]), we selected a few varieties of the IMPROMALT population of barley [32] that seemed to be either tolerant or more sensitive to waterlogging in the field [31] and then used them to compare two different methods.

## Materials and methods

### Plant material

The five cultivars used in this study originated from the IMPROMALT population of barley [32] and included 2-row varieties (Cavalier and KWS Infinity), as well as 6-row varieties (Dura, Isa, and Siberia).

### Plant growth conditions

For partial submergence experiments with a starch solution, plants were grown in growth chambers (Snijders Micro Clima High Specs Plant Growth Cabinet MC1750E and Snijders Micro Clima MC1204) programmed with the following parameters: 12 h dark at 7 °C with 80% relative humidity and 12 h light (~ 138 µmol/m^2^/s; provided by LED lighting Quictronic OT 1 × 58 W) at 14 °C with 80% relative humidity. The latter growth conditions were chosen to simulate the average outdoors conditions observed on-site (Oak Park, Co. Carlow, Ireland) during the month of October (Additional file 1: Table S1).

For partial submergence experiments with water, plants were grown in a custom-made plant growth room under long-day conditions (16 h light/8 h dark) at 15 °C (constant temperature), approximately 45% relative humidity. Light intensity was determined to be ~ 138 µmol/m^2^/s and was provided by LED bulbs (Philipps LED tubes High Output, T8 20 W/865).

### Soil type and preparation

For partial submergence experiments with a starch solution, a custom-made soil mix that resembles field soil was used (Westland Horticulture; Ireland): 80% (v/v) sterilised loam, 19.5% (v/v) 3–6 mm lime-free grit and 0.4% (v/v) Osmocote mini. The soil was soaked before potting to prevent soil loss from drainage holes. Each pot (6 × 6x7 cm; LxWxH) was filled with 210 g of wet soil.

For partial submergence experiments in water, commercial John Innes N^o^2 (Vitax; UK) soil was soaked in water after filling round pots of 9-cm diameter and 9-cm height without compressing the soil.

### Seed germination

For partial submergence experiments with a starch solution, all seeds were coated with fungicide (Redigo Deter™ at a concentration of 2 µL/g of seeds). All seeds were then germinated in the dark (80% relative humidity; 14 °C) on filter paper imbibed with water for four days before planting in soil and grown as indicated in Plant Growth Conditions. The seeds were not stratified prior to germination.

For partial submergence experiments in water, untreated seeds were sown directly in soil (John Innes N^o^2) at a depth of 2 cm. The sown seeds were stratified in the dark for 14 days at 4 °C to ensure homogenous germination. After cold treatment, the pots were transferred to the plant room for germination and growth (see growth conditions above).

### Partial submergence treatment with a 0.1% starch solution

This protocol was adapted from Mano and Takeda [22]. The plants were grown for 15 days (two visible leaves), after which half of the pots were transferred to a large container filled with a 0.1% (w/v) starch (Sigma-Aldrich) solution prepared in deionised water. Typically, 5 L of the starch solution were used until the water level reached 1 cm above the soil (tray dimensions: 31 × 18 × 8 cm; L × W × H; 15 pots per tray). The water level was measured every two days and topped up with 0.1% starch solution as needed. The plants were kept in the same growth conditions as indicated above, and treatment was applied for 15 days, or for shorter periods of time, as indicated in the text. Control plants were left in the same growth conditions but received normal watering (watering every two days, avoiding any standing water in the trays). For the recovery period, plants were taken out of the 0.1% starch solution and kept in the same growth conditions with a normal watering regime.

### Partial submergence with water

To apply partial submergence with water, the plants were grown for ten days (at which point, two leaves had emerged) and then the pots were transferred to a large tub, which was subsequently filled with tap water up to 1 cm above soil level. The water level was kept constant throughout the duration of the experiment. The plants were kept in the same growth conditions and were treated for 15 days, or for shorter periods of time, as indicated in the text. Control plants were left in the same growth conditions but received normal watering (watering every two days, avoiding any standing water in the trays). For the recovery period, plants were taken out of the water and kept in the same growth conditions with a normal watering regime.

### Total RNA extraction

Roots of plants grown under control conditions or subjected to partial submergence with either water or a 0.1% starch solution for 24 h were rinsed and frozen in liquid nitrogen. The tissue was ground in liquid nitrogen and total RNA was extracted using Spectrum™ Plant Total RNA kit (Sigma-Aldrich).

### Reverse transcription coupled to quantitative PCR (RT-qPCR)

Total RNA was reverse transcribed using an oligo(dT)_18_ primer and the RevertAid reverse transcriptase (Thermo) according to the manufacturer’s instructions. For reverse transcription reactions with samples obtained from experiments with water partial submergence, 1 µg of total RNA was used, while for samples obtained from experiments with the starch solution, 0.4 µg of total RNA was used. cDNA obtained was used for qPCR with a Lightcycler 480 (Roche). Each PCR reaction mix contained 5 μL of 2 × SYBR green master 1 (Roche), 1 μL cDNA, 1 μL of 10 μM primers and 3 μL of molecular biology grade water. LightCycler melting curves were obtained to check for single peak melting curves for all amplification products. The second derivative maximum method was used to analyze the amplification data. The resulting Cp values were converted into relative expression values using the comparative Ct method [33]. One reference gene (Hv*ACTIN*) was used to normalize the data. Oligonucleotides used were as follows (indicated 5′ to 3′): H*vADH1* (HORVU4Hr1G016810)—HvADH1F (CACTGACCTGCCCAATGTC) and HvADH1R (GCACGCTGTGTGTGATGAA) [27]; *HvHB* (HORVU4Hr1G066200)—AM51 (CGGGAAGGAAGCCATGTCTGC) and AM52 (TCTGCCTCGCCGACGG); Hv*ACTIN* (HORVU1Hr1G002840)—AM45 (GCAAGTGGTCGTACTACTGGTATCGTTC) and AM46 (GGATCTTCATAAGGGAGTCCGTGAGAT).

### Height measurements

Plant height was taken from the soil surface to the tip of the tallest leaf at the indicated time points.

### Chlorophyll content determination

The chlorophyll content was determined using two methods. First, the soil–plant analysis development (SPAD) values were determined using a hand-held SPAD meter (Minolta SPAD meter 502). The measurement was made 1 cm from the tip of leaf 1. In addition, total chlorophyll was extracted from 5 mg of leaf tissue (partial submergence with water) or the 1-cm tip of the oldest leaf (starch treatment) in 1 mL of 80% (v/v) acetone as described by Sumanta et al. [34]. Chlorophyll a (chl a) and chlorophyll b (chl b) content was measured based on the absorbance at 646 nm (A_646_) and 663 nm (A_663_), respectively. The equations used to calculate the amount of chlorophyll were:

$${\text{chla }} = { 12}.{\text{25 A}}_{{{663}}} {-}{ 2}.{\text{79 A}}_{{{646}}}$$

$${\text{chlb }} = { 21}.{\text{5 A}}_{{{646}}} {-}{ 5}.{\text{1 A}}_{{{663}}} .$$

### Phenotypic scoring

At the time points specified during the recovery period, each plant was photographed and/or assessed visually to assign a score as detailed in Table 1 and in Additional file 1: Figure S1.

**Table 1:** Criteria to score plants after partial submergence in water or in a 0.1% starch solution

Score	Leaf colour	Leaf wilting
1 (sensitive)	All leaves show discolouration	All Leaves are wilted and show signs of necrosis
2	75% of leaves show discolouration	All Leaves are wilted
3	50% of leaves show discolouration	Some wilting
4	Slight discolouration at tips of leaves	Some wilting
5	Slight discolouration at tips of leaves	No wilting
6 (tolerant)	All leaves are green	No wilting

Pictures illustrating each of these criteria are shown in Additional file 1: Figure S1

### Statistic tests applied

Differences between varieties and treatments were tested using an unpaired Student’s t-test and Welch correction because of unequal sample size and variation. *P*-values obtained are indicated in the figure legends.

## Results

### Description of experimental setups and differences

**Fig. 1:**
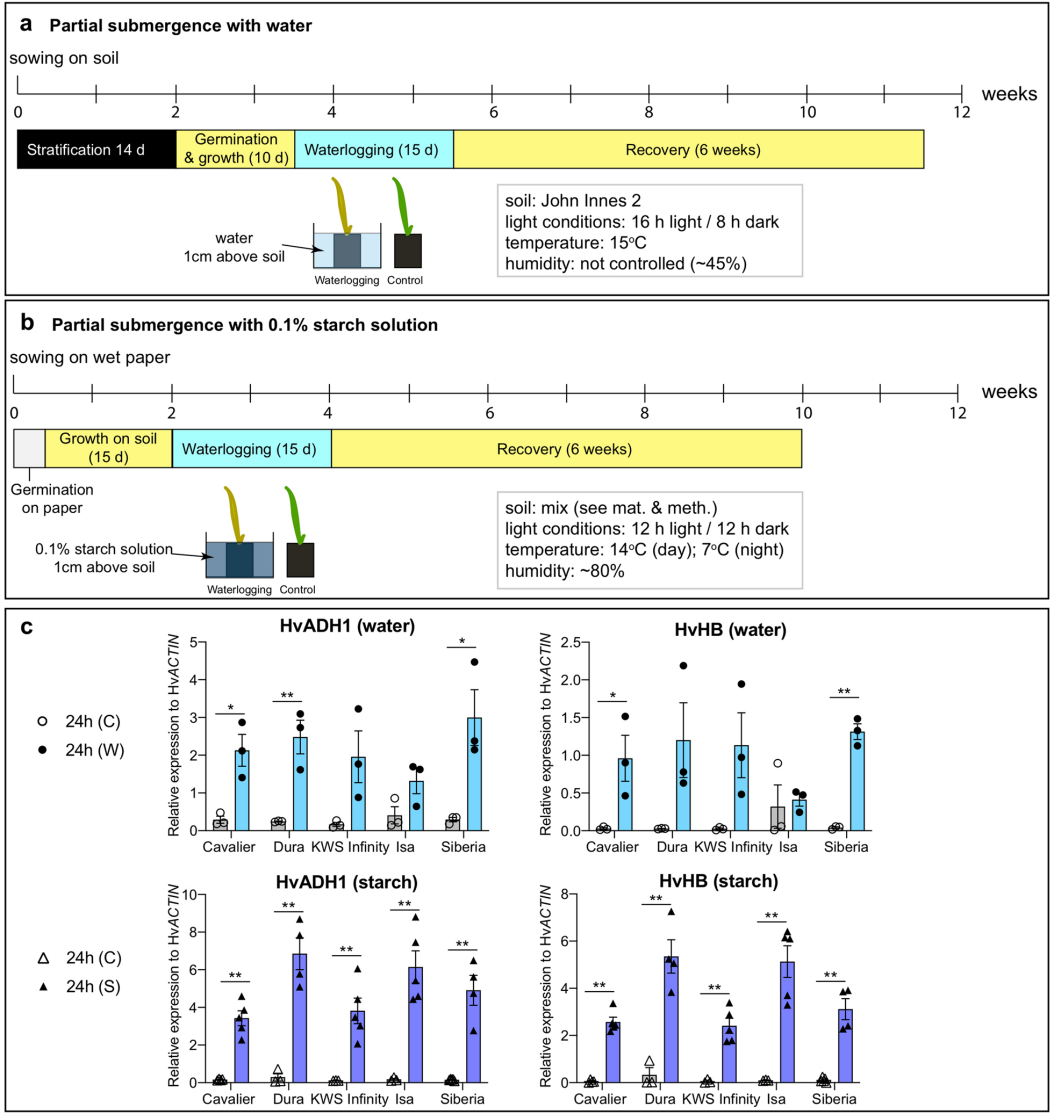
Overview of the growth conditions used in this study. **a** Growth conditions when applying partial submergence with water. Plants were grown for 10 days (at which point, two leaves had emerged). Partial submergence was applied by transferring the pots to a large tub filled with tap water 1 cm above soil level. The plants were kept in the same growth conditions during the duration of the experiment. Control plants were left in the same growth conditions but received normal watering. For the recovery period, plants were taken out of the water and kept in the same growth conditions with a normal watering regime. **b** Experimental conditions used when partial submergence with a 0.1% starch solution was applied. This protocol was adapted from Mano and Takeda [22]. The plants were grown for 15 days (two visible leaves), after which half of the pots were transferred to a large container filled with a 0.1% (w/v) starch solution, with solution reaching 1 cm above soil level. The plants were kept in the same growth conditions. Control plants were left in the same growth conditions but received normal watering. For the recovery period, plants were taken out of the 0.1% starch solution and kept in the same growth conditions with a normal watering regime. Detailed experimental conditions are provided in Materials and Methods. **c** Gene expression changes for two core hypoxia response genes (Hv*ADH1* and Hv*HB*) under control conditions (24 h (C)) and after 24 h of partial submergence with either water (24 h (W)) or a 0.1% starch solution (24 h (S)). Average expression relative to that of the reference gene (Hv*ACTIN*) is shown (3 to 4 independent replicates were carried out; 3 roots pooled *per* replicate and conditions). Error bars correspond to standard error of the mean. Significant statistical differences (determined using Student’s t-test) are indicated with: 0.01; * for p-value < 0.05

Two protocols to screen for barley waterlogging tolerance were tested to assess and compare their effects on plant growth and health (Fig. 1a, b). All seeds used were derived from the same seed batch to reduce the potential effects of different seed batches on the experiments. For the first protocol, which included partial submergence with water, barley was grown under long days and constant temperature of 15 °C, on a rich commercial potting medium (John Innes N°2). The second protocol, which included partial submergence with a 0.1% (w/v) starch solution, was set up to take into account growth conditions that are close to those encountered in the field, including a custom-made soil mix, seed coating and growth conditions with day/night temperature variations and higher humidity that mimicked those of the natural conditions on site (Oak Park, County Carlow, Ireland). Importantly, it has been shown that the use of a 0.1% starch solution induces reducing soil conditions (or low soil redox potential) [22], which often accompany waterlogging or (partial) submergence in the wild and trigger additional chemical changes in the soil [35].

The partial submergence treatments applied are expected to trigger the onset of the low-oxygen (hypoxia) response program, including the up-regulation of core hypoxia-response genes such as those coding for alcohol dehydrogenase (Hv*ADH1*) and hemoglobin (Hv*HB*). In order to verify that the two partial submergence treatments applied indeed resulted in hypoxia, we determined using RT-qPCR the gene expression changes of Hv*ADH1* and Hv*HB* after 24 h of partial submergence treatment. Our results show that these two genes were up-regulated upon partial submergence with either water or a 0.1% starch solution, thus confirming that the conditions used were suitable to induce hypoxia and study the effect of partial submergence on barley plants (Fig. 1c). The up-regulation of Hv*ADH1* and Hv*HB* in the different varieties tended to be higher upon treatment with the 0.1% starch solution compared to water, possibly suggesting that the use of a 0.1% starch solution results in a stronger stress and response.

### Partial submergence with a 0.1% starch solution affects chlorophyll content and nitrogen use efficiency more strongly than partial submergence with water

To assess plant tolerance to waterlogging or partial submergence, the amount of chlorophyll in leaf tissue is measured, with higher chlorophyll levels being associated with a better tolerance to the stress. Chlorophyll content also tends to correlate with nitrogen (N) use efficiency [36], which is particularly relevant in the context of partial submergence or waterlogging, because of the resulting reduced nitrate availability [7].

**Fig. 2:**
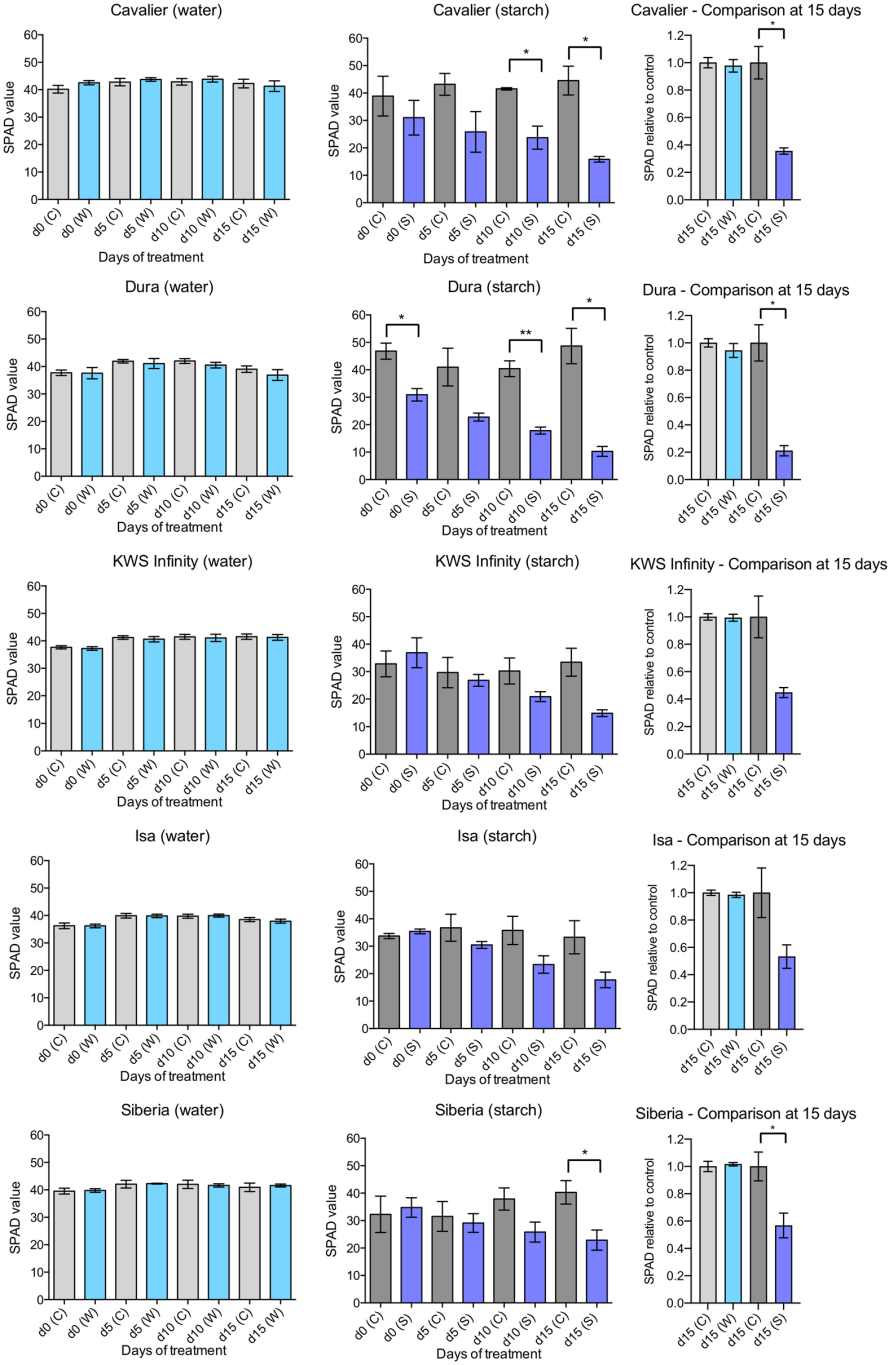
Effect of partial submergence with water or a 0.1% starch solution on chlorophyll content. SPAD values for leaf 1 were determined using a hand-held SPAD meter. Data on the left was obtained for plants subjected to partial submergence with water or control conditions. Three independent replicates were performed, with four plants per replicate for each condition. Data in the centre was obtained when partial submergence was applied using a 0.1% starch solution or under control conditions. Three independent replicates were performed, with the number of plants used per replicate varying between one and three. Data on the right represent SPAD values after partial submergence relative to the SPAD values obtained with control plants for a given variety. Mean values and standard error of the mean for the three replicates are shown. Statistical significance of the differences was determined using a Welch’s t-test because of unequal variance and sample sizes. Significant statistical differences are indicated with: ** for p-values $<$ 0.01; * for p-value $<$ 0.05

For both partial submergence protocols tested, a handheld SPAD meter was used to determine chlorophyll content. Because this method is not destructive, changes in the SPAD values were followed at regular intervals (every 5 days) from the onset of the partial submergence treatment until the end of the experiment (i.e. after 15 days of partial submergence). Measurements were conducted on leaf 1, which was already formed at the beginning of the treatment. When partial submergence with water was applied, the SPAD values obtained for the five different varieties did not vary between the beginning of the experiment (day 0) until the end of the treatment at day 15 (Fig. 2). In addition, there were no differences in SPAD values between the control plants and those subjected to partial submergence (all varieties tested had similar SPAD values comprised between 35 and 40 in average). Hence, the SPAD values obtained suggest that chlorophyll content was not affected by the treatment and that N use efficiency was maintained.

Under the experimental conditions used to test partial submergence with a 0.1% starch solution, the SPAD values obtained for the control plants were within the same range as those measured for the plants used for partial submergence in water, suggesting that the different growth conditions did not affect significantly chlorophyll content and overall N use efficiency (Fig. 2). In addition, for all varieties tested, the SPAD values measured for the control plants did not vary significantly between day 0 and day 15. In contrast, the SPAD values obtained following the onset of partial submergence with a 0.1% starch solution decreased. This decrease was typically significant at days 10 and 15 of the treatment, and was observed in most varieties except Isa, for which differences were not statistically significant due to variation between replicates. One exception is the lower SPAD values obtained for Dura at day 0, which is likely due to variation within each of the populations tested. This difference makes the interpretation of the differences at day 10 and 15 more difficult for Dura. The stronger effect of the 0.1% starch solution was confirmed when plotting, for each variety, the relative SPAD values of treated plants compared to those of control plants after 15 days of partial submergence (Fig. 2).

To complement our results, after 15 days of treatment, the leaves used for SPAD measurements were collected to extract and estimate the content of chlorophyll a (chl a), chlorophyll b (chl b) and carotenoid. The ratio of chl a/b was then calculated, as this ratio reflects (i) N availability (higher chl a/b ratios are associated with a decrease in N availability [36]), and (ii) photosynthetic capacity. Similarly to the results obtained with the SPAD meter, partial submergence with water did not alter the chl a/b ratio of the different varieties (Additional file 1: Figure S2). The chl a/b ratio was also similar for all varieties tested. For the plants subjected to partial submergence with a 0.1% starch solution, the values obtained showed more variation, possibly as a result of (i) the lower number of plants used to extract photosynthetic pigments, and (ii) the tissue collection method which resulted in more variable and lower amounts of tissue for each sample, making the interpretation of the data more difficult (Additional file 1: Figure S2). Although not statistically significant, for several of the varieties tested, there seemed to be a slight decrease in the chl a/b ratio.

In sum, although the different growth conditions did not affect chlorophyll content and N use efficiency for the control plants, treatment with a 0.1% starch solution had a stronger effect on the loss of chlorophyll and N use efficiency than partial submergence with water alone.

### Partial submergence with a 0.1% starch solution has a stronger effect on plant wilting and yellowing

**Fig. 3:**
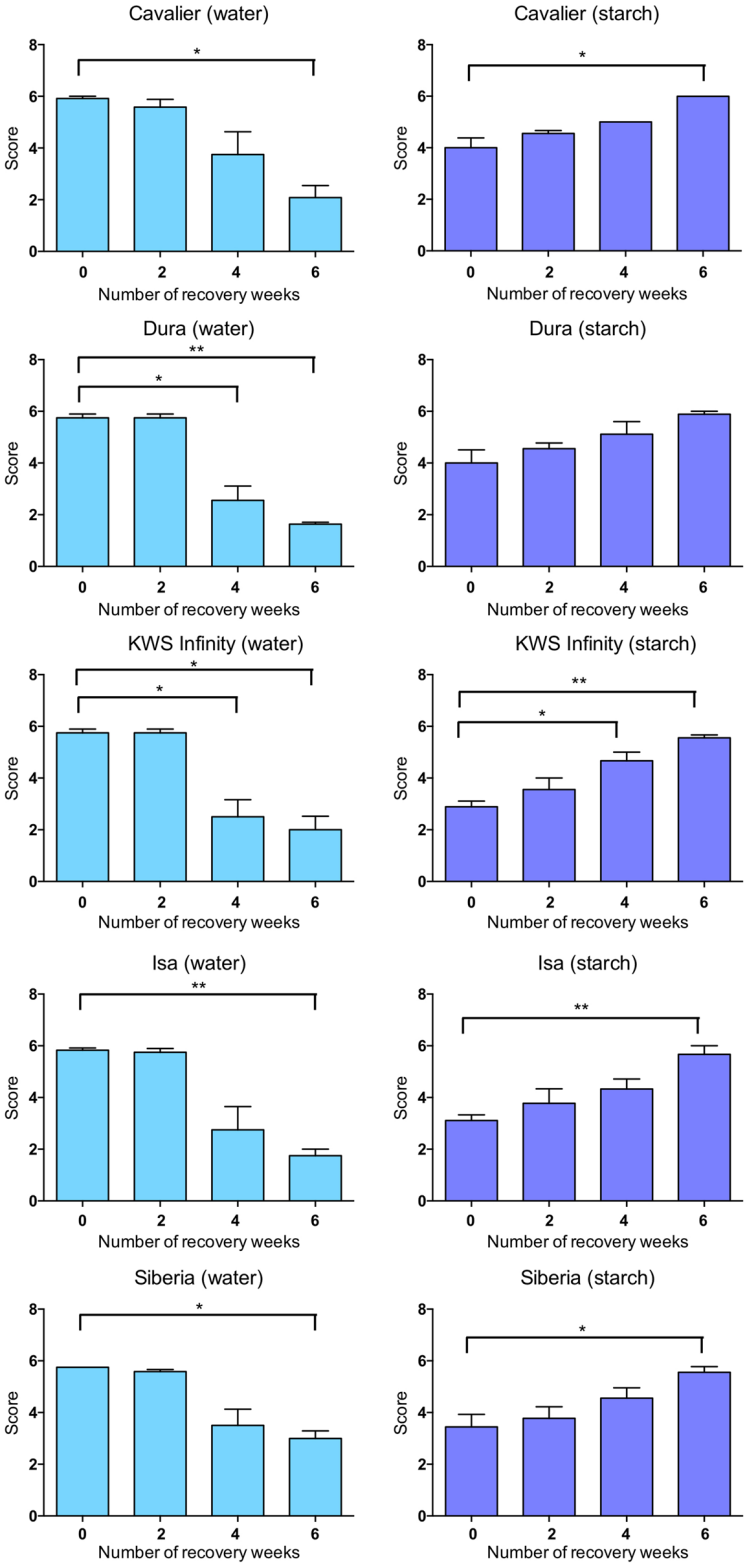
Average scores during recovery after partial submergence with water or with a 0.1% starch solution. Data on the left was obtained for plants subjected to partial submergence with water. Three independent replicates were performed, with three to four plants per replicate for each condition (with the exception of the 2-week time point, for which only one plant was used in one of the replicates with Cavalier and Dura). Data on the right was obtained when partial submergence was applied using a 0.1% starch solution. Three independent replicates were performed, with three plants per replicate (with the exception of Dura, for which only two plants were used in one of the replicates). Mean values and standard error of the mean for the three replicates are shown. Significant statistical differences (determined using a Welch’s t-test) are indicated with: ** for p-values $<$ 0.01; * for p-value $<$ 0.05

In order to assess the tolerance/sensitivity of the five varieties using a wider range of criteria, a scoring system was established by combining different vegetative traits that are affected by hypoxia stress, including chlorosis, necrosis and wilting [23]. Based on these traits, scores ranging from 1 (plants severely affected by stress) to 6 (plants remaining healthy despite partial submergence) were assigned to each of the varieties tested, as outlined in Table 1 and Additional file 1: Figure S1. Importantly, because the scoring relies on visual assessment and does not involve destructive methods, it allowed us to monitor the same plants at different time points from the end of the partial submergence treatment until 6 weeks after they were returned to a normal watering regime (i.e. during the recovery period). Although plants that had experienced partial submergence with water appeared to be unaffected after 15 days of treatment (as indicated as well by their SPAD and chl a/b values), their overall health decreased during the recovery period, with scores decreasing from approximately 6 to 2 in average within the 6 weeks of recovery (Fig. 3). This result suggests that partial submergence with water has long-term effects on barley, and that the impact of the treatment becomes visible later in development, around 4 weeks after the end of the stress, even though the plants had returned to a normal watering regime. In contrast, while plants experiencing partial submergence with a 0.1% starch solution were severely affected immediately after 15 days of treatment, they appeared to recover well when returned to normal watering, suggesting that the effects of the 0.1% solution were transient (Fig. 3).

In sum, the scoring system established allowed us to monitor overall plant health after partial submergence and during a 6-week recovery period. Our results show that treatment with water or with a 0.1% starch solution have very different effects on the plants and how they recover.

### Differential effects of the partial submergence treatments on height during recovery

**Fig. 4:**
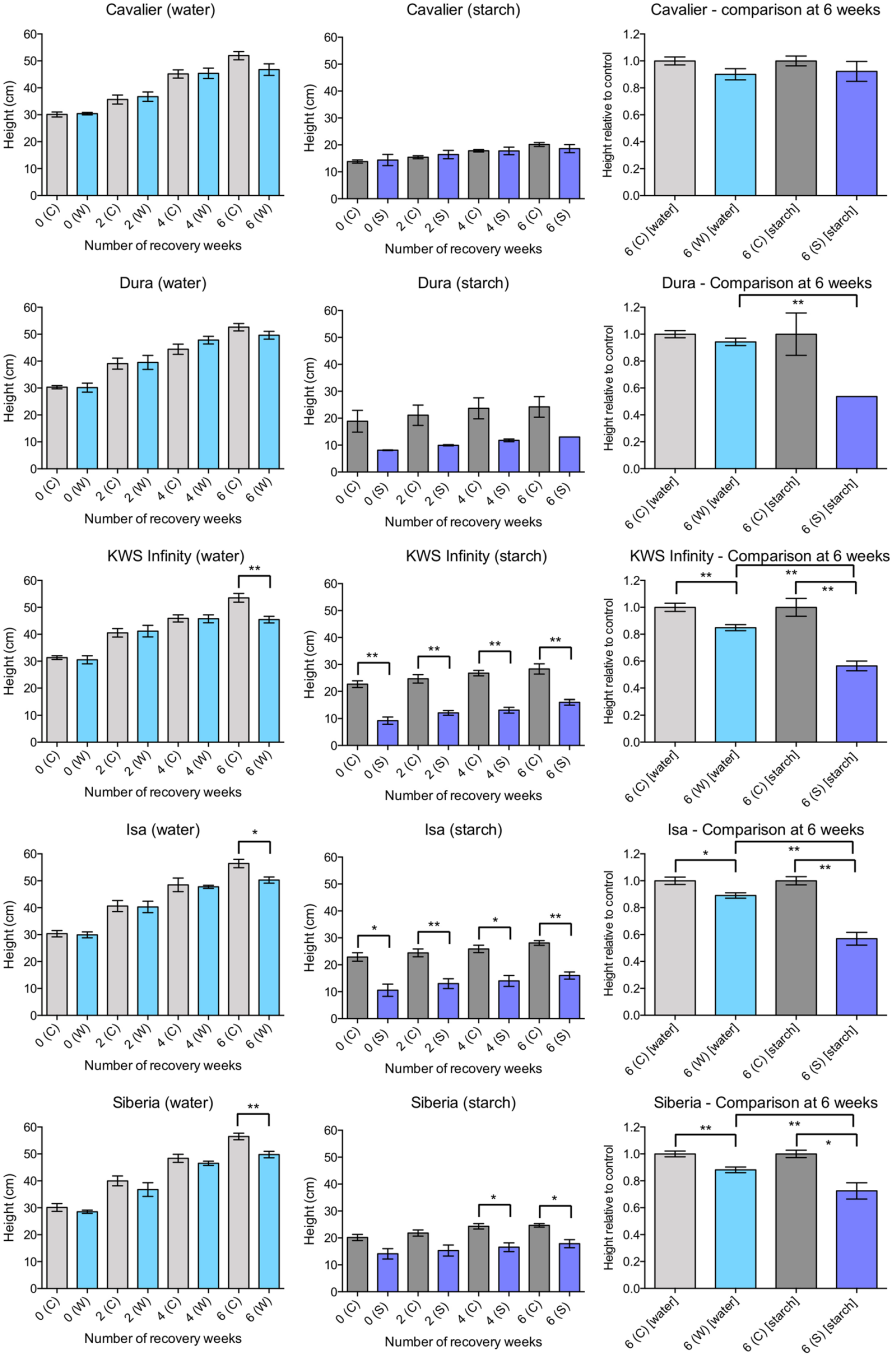
Average plant height during recovery after partial submergence with water or with a 0.1% starch solution. Data on the left was obtained for plants subjected to partial submergence with water or control conditions. Four independent replicates were performed, with three to four plants per replicate for each condition. Data in the centre was obtained when partial submergence was applied using a 0.1% starch solution or under control conditions. Three independent replicates were performed, with two to three plants per replicate for the 0.1% starch treatment. For control conditions with Siberia, the data is representative of two independent replicates with three plants per replicate. Mean values and standard error of the mean for the three replicates are shown. Data on the right represent plant height after partial submergence relative to the height of control plants for a given variety. Significant statistical differences (determined using a Welch’s t-test) are indicated with: ** for p-values $<$ 0.01; * for p-value $<$ 0.05

The height of each plant was also measured at day 0 of the recovery period (i.e. after 15 days of partial submergence or control treatment), and at 2, 4 and 6 weeks of recovery, with the idea that varieties whose height was not affected compared to control plants could be considered as more tolerant. The same plants that were scored were used for the height measurements. For up to 4 weeks after partial submergence with water, control and treated plants had similar height, suggesting again that the treatment had no immediate effect on plant growth (Fig. 4). For three varieties—KWS Infinity, Isa and Siberia—the height of plants subjected to partial submergence with water was reduced compared to that of the control plants, but this difference only appeared late in the recovery period, at 6 weeks. The other two varieties tested, Cavalier and Dura, showed no significant differences in height between control and treated plants (Fig. 4).

Height changed differently in plants that experienced partial submergence with a 0.1% starch solution. For the three varieties whose height was affected when treated with water (KWS Infinity, Isa and Siberia), statistically significant differences were observed at day 0 of the recovery and throughout the 6 weeks of recovery (Fig. 4). These results suggest again that treatment with a 0.1% starch solution has a strong effect on plant health and growth during the 15 days of treatment. Cavalier, a variety whose height was not affected by partial submergence in water, was also unaffected for this trait in the presence of 0.1% starch, even after 6 weeks of recovery. Dura also appeared to have a reduced height overall, but the differences were not statistically significant because of variability.

Notably, the growth conditions alone seemed to have an impact on plant height. For a given variety, height was typically lower when plants were grown under the conditions used to test the effects of a 0.1% starch solution (Fig. 4). To compare more directly the effects of the two partial submergence treatments, for each variety, the relative height of treated plants compared to that of control plants was calculated at 6 weeks of recovery (Fig. 4). The comparison confirmed that the two methods affect height differently, with 0.1% starch treatment having a stronger effect on plant height than water. Notably, though, the two methods gave qualitatively similar results, with slightly stronger effects after treatment with a 0.1% starch solution at earlier time points.

### Differential effects of the partial submergence treatments on tiller numbers during recovery

**Fig. 5:**
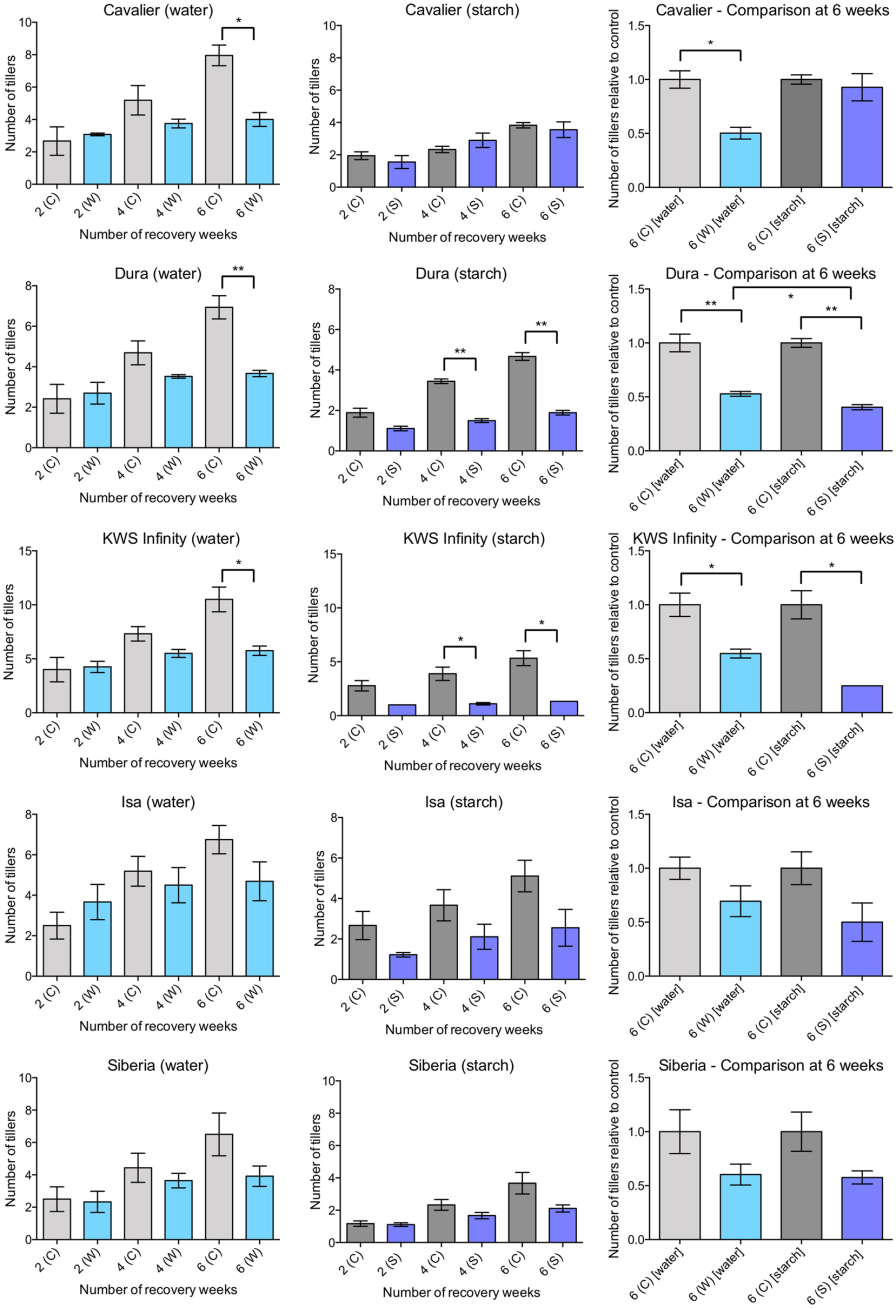
Average number of tillers during recovery after partial submergence with water or with a 0.1% starch solution. Data on the left was obtained for plants subjected to partial submergence with water or control conditions. Four independent replicates were performed, with three to four plants per replicate for each condition. Data in the centre was obtained when partial submergence was applied using a 0.1% starch solution or under control conditions. Three independent replicates were performed, with two to three plants per replicate for the 0.1% starch treatment. For control conditions with Siberia, the data is representative of two independent replicates with three plants per replicate. Mean values and standard error of the mean for the three replicates are shown. Data on the right represent the number of tillers after partial submergence relative to the number of tillers of control plants for a given variety. Statistical significance of the differences was determined using a Welch’s t-test. Significant statistical differences are indicated with: ** for p-values $<$ 0.01; * for p-value $<$ 0.05

Because of its direct relevance to yield, the number of tillers was also determined at 2, 4 and 6 weeks after the end of a 15-day partial submergence treatment, or in plants kept under control conditions. After treatment with water, the number of tillers between control and partially submerged plants were similar, but differences appeared around 4 weeks of recovery and, in most cases, became statistically significant after 6 weeks of recovery (Fig. 5). Specifically, although plants kept under control conditions had increased number of tillers throughout the 6 weeks of recovery period, plants subjected to partial submergence with water did not appear to form more tillers than those visible at 2 weeks of recovery. As a result, partially submerged plants in water produced in average fewer tillers than control plants.

When plants were partially submerged in a 0.1% starch solution, the number of tillers appeared to be somewhat reduced already at 2 weeks of recovery for some varieties such as Dura, KWS Infinity or Isa. Similarly to the observations made after partial submergence in water, the number of tillers in plants partially submerged in starch solution seemed to produce fewer tillers than plants that had been kept under control conditions (Fig. 5). An exception was Cavalier, which seemed to be unaffected for tiller numbers, as was the case for plant height.

To compare the effects of the two methods on tiller number and reduce the effect of variety-specific differences or of the different growth conditions, the relative tiller number of treated plants compared to control plants was calculated at 6 weeks (Fig. 5). The comparison suggested that, in most cases, treatment with a 0.1% starch might have a stronger effect on tiller number than treatment with water. One notable exception is Cavalier, which seemed largely unaffected after partial submergence with a 0.1% starch solution, while partial submergence with water had detrimental effects. Nevertheless, the two methods gave overall qualitatively similar results.

## Discussion

The comparison of different barley varieties to waterlogging or (partial) submergence stress is difficult, not only because of the complex nature of the stress and of the genotypic plant responses to it, but also because there are no standardized protocols available. In different studies, plants are grown in different conditions and soils, the stress is applied at diverse developmental stages and different criteria are applied for scoring. Here, we have compared two methods in controlled conditions that can be used to characterize barley responses to waterlogging or (partial) submergence and rank varieties. We specifically focused on winter barley varieties because they are sensitive to waterlogging or (partial) submergence and applied the treatments at early stages of development, as could occur in winter [7, 37].

The first protocol included the use of a rich commercial compost, long days and constant temperatures of 15 °C. As in many other studies, partial submergence was applied using water. The second protocol tested involved partial submergence with a 0.1% starch solution using plants grown under conditions that mimicked those found in temperate climate in autumn or early winter. The use of a 0.1% starch solution allowed to decrease efficiently soil redox potential, as would typically occur under field conditions. This method had been previously used to rank barley varieties [22]. Overall, the two protocols produced different responses in plants. Plants treated with a 0.1% starch solution started yellowing within five days of treatment and were severely affected after 15 days of partial submergence. Notably, though, upon return to a normal watering regime, most varieties recovered, so that after 6 weeks of recovery, most varieties had a score close to 6 (Fig. 3). This potential recovery may be due to the fact that the new leaves formed were healthy after returning to normal watering. Nevertheless, the effect of the treatment persisted, as plant growth was stunted and a lower number of tillers formed. In contrast, plants that experienced partial submergence with water appeared to be largely unaffected during the 15 days of treatment (i.e. SPAD values, chlorophyll levels and scores were not significantly different from those of control plants). However, within 4 to 6 weeks of recovery, treated plants showed reduced height and a lower number of tillers, indicating that partial submergence with water had in fact a long-term effect on the plants. The different effects of the two protocols may be related to the treatments or to differences in soil composition and/or night-time temperature. Another plausible explanation for the differences observed between the treatments is that the shorter 12-h photoperiod used when applying the 0.1% starch solution (as opposed to 16 h light/8 h dark with water treatment) could contribute to starvation, thus possibly enhancing the effects of the partial submergence treatment with the starch solution. To address a possible effect of day length on barley tolerance to the two partial submergence treatments, we repeated the SPAD and height measurements, as well as scoring of the plants, after 15 days of partial submergence with reversed photoperiod conditions. Specifically, the starch solution was applied to plants grown under 16 h light/8 h dark at 15 °C, while the water treatment was applied to plants grown under 12 h light/12 h dark at 15 °C. Our results show that the shorter photoperiod combined with the starch solution indeed produced a stronger phenotypic difference in barley plants compared to a longer photoperiod combined with the starch solution (Additional file 1: Figure S3). In contrast, the effects of partial submergence with water were not affected by the photoperiod used (Additional file 1: Figure S3). These experiments thus highlight the importance of using a shorter photoperiod, especially if working with a 0.1% starch solution for partial submergence.

**Fig. 6:**
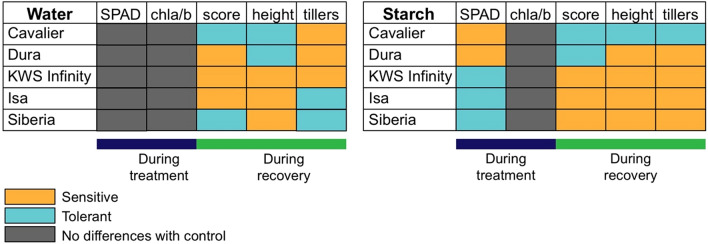
Ranking of barley varieties based on the two methods applied. Based on each of the traits monitored, varieties that seemed to be the least affected by the partial submergence treatments compared to control plants of the same variety were classified as tolerant (blue). In contrast, varieties that were more strongly negatively affected by the partial submergence were classified as sensitive (orange). Traits for which no significant differences could be detected between control and treated plants are indicated in grey. SPAD and chl a/b measurements were performed during the partial submergence treatment (indicated by dark purple line underneath), while other traits were monitored during the recovery period (highlighted by green line). Note that for the ‘score’ category, ranking was done at day 0 of the recovery for treatment with 0.1% starch, while for partial submergence for water, the scores at four and 6 weeks of recovery were taken into account

Using each of the parameters, we attempted to classify the plants as more or less tolerant to partial submergence (Fig. 6). Within a given treatment, the results were not consistent across all the traits monitored, although partial submergence with a 0.1% starch solution seemed to produce overall more consistent data across the different traits scored, as suggested previously [22]. Nevertheless, when comparing qualitatively the results, there was some consistency across the two methods, in that Cavalier appeared to be more tolerant to partial submergence, while KWS Infinity and Isa seemed to be more sensitive. Conclusions for Dura and Siberia were more difficult to draw because of variability for each of the methods among different traits scored, or because results obtained using the two methods differed. In sum, both methods are complementary and can be used in parallel for a more robust assessment of barley tolerance/sensitivity to partial submergence. The use of a 0.1% starch solution can provide some useful information at earlier stages of the experiment (i.e. during the partial submergence treatment), but it remains important to characterize plant responses during the recovery period. The latter is indeed crucial in the field and is also known to be a source of stress for plants.

## Supplementary Information

**Additional file 1: Table S1.** Five-year weather temperature and precipitation averages for the month of October in Oak Park (Co. Carlow, Ireland). **Figure S1.** Representative pictures of plants for the different scores outlined in Table 1. Pictures taken after 15 days of partial submergence with a 0.1% starch solution. **Figure S2.** Chlorophyll content in control plants and after partial submergence treatment. **Figure S3.** Effect of photoperiod of the phenotypic differences upon partial submergence.

## Data Availability

The datasets during and/or analysed during the current study available from the corresponding author.
