# Supplementary material for: Experimental comparison of two methods to study barley responses to partial submergence
Source: Plant Methods. 2021 Apr 13;17:40. doi: 10.1186/s13007-021-00742-5 (PMC8045378; doi:10.1186/s13007-021-00742-5)
Supplement: Supplementary file 1 — Additional file 1: Table S1. Five-year weather temperature and precipitation averages for the month of October in Oak Park (Co. Carlow, Ireland). Figure S1. Representative pictures of plants for the different scores outlined in Table 1. Pictures taken after 15 days of partial submergence with a 0.1% starch solution. Figure S2. Chlorophyll content in control plants and after partial submergence treatment. Figure S3. Effect of photoperiod of the phenotypic differences upon partial submergence. [file 13007_2021_742_MOESM1_ESM.docx]

**Supplemental Information**

**Experimental comparison of two methods to study barley responses to partial submergence**

Alexandra Miricescu^1#^, Tomás Byrne^3#^, Catherine M. Doorly^1,2^, Carl KY Ng^4*^, Susanne Barth^3*^ and Emmanuelle Graciet^1,2*^

^1^ Maynooth University, Department of Biology, Maynooth, Co. Kildare, Ireland

^2^ Kathleen Lonsdale Institute for Human Health Research, Maynooth University, Maynooth, Co. Kildare, Ireland

^3^ Teagasc Crops, Environment and Land Use Program, Crop Science Department, Oak Park, Co.Carlow, R93XE12, Ireland

^4^ University College Dublin, School of Biology and Environmental Science, Centre for Plant Science, UCD Earth Institute, O’Brien Centre for Science West, Belfield, Dublin, D04 N2E5, Ireland

# equal contribution

* corresponding authorship

**Table S1: Five-year weather temperature and precipitation averages for the month of October in Oak Park (Co. Carlow, Ireland).** Meteorological details were collected at the Oak Park weather station. Meant: mean air temperature (°C); Maxtp: maximum air temperature (°C); Mintp: minimum air temperature (°C); Mnmax: mean maximum temperature (°C); Mnmin: mean minimum temperature (°C); rain: precipitation amount (mm). Data available at: <https://www.met.ie/climate/available-data/historical-data>.

| Year | Meant (°C) | Maxtp (°C) | Mintp (°C) | Mnmax (°C) | Mnmin (°C) | rain (mm) |
| --- | --- | --- | --- | --- | --- | --- |
| 2015 | 10.2 | 19.1 | 0.3 | 14.2 | 6.2 | 56.8 |
| 2016 | 10.6 | 16.6 | 2.1 | 14.4 | 6.8 | 32.3 |
| 2017 | 11.5 | 18.8 | 1 | 14.9 | 8.1 | 62.9 |
| 2018 | 10 | 18.7 | -2.3 | 13.8 | 6.1 | 58.3 |
| 2019 | 9.4 | 17.7 | -0.8 | 13.2 | 5.7 | 102.3 |
| 5-year average | 10.3 | 18.2 | 0.06 | 14.1 | 6.58 | 62.52 |


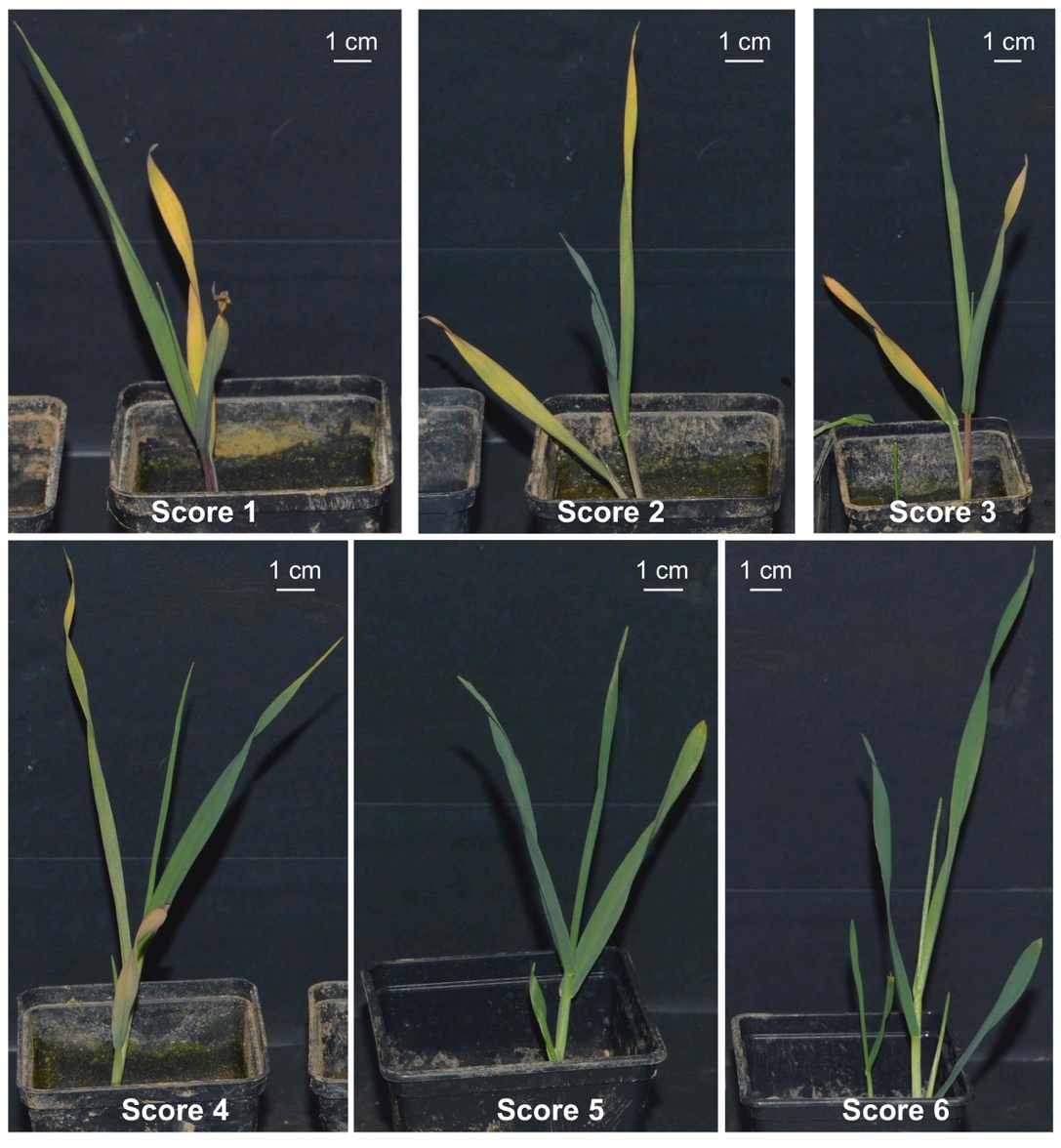


**Figure S1: Representative pictures of plants for the different scores outlined in Table 1.** Pictures taken after 15 days of partial submergence with a 0.1% starch solution.


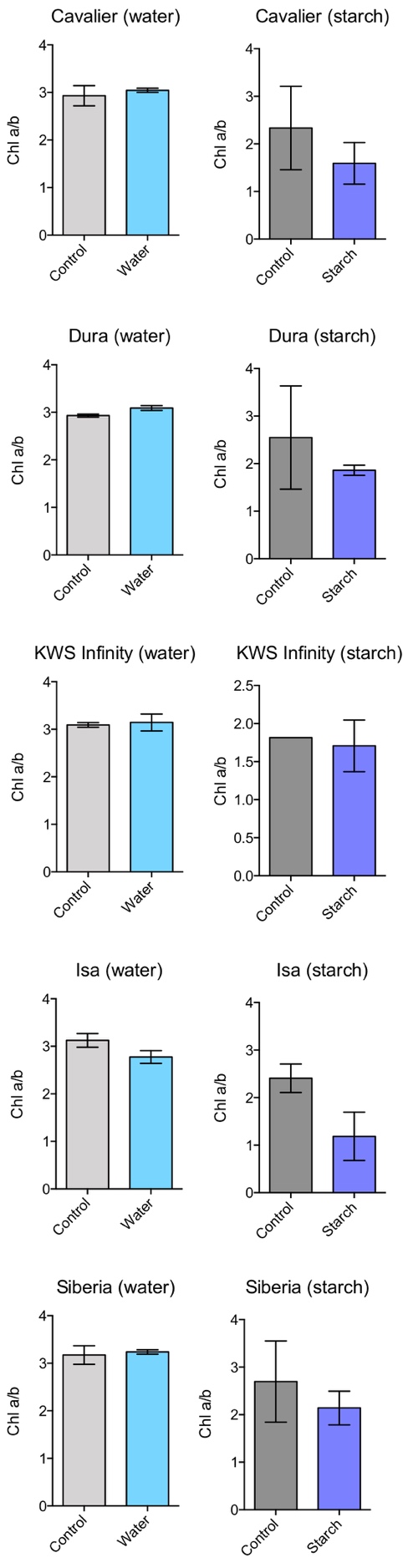


**Figure S2: Chlorophyll content in control plants and after partial submergence treatment.** Chl a/b ratios for leaf 1 were determined following pigment extraction in 80% acetone. Data on the left was obtained for plants subjected to partial submergence with water or control conditions. Three independent replicates were performed, with 3 to 4 plants per replicate for each condition. Data on the right was obtained when partial submergence was applied using a 0.1% starch solution or under control conditions. Three independent replicates were performed, with 3 plants per replicate. For Infinity, only one replicate was available for the control conditions (3 plants). Mean values and standard error of the mean for the 3 replicates are shown. Statistical significance of the differences was determined using a Welch’s t-test because of unequal variance and sample sizes. None of the differences were statistically significant (p-values > 0.05).


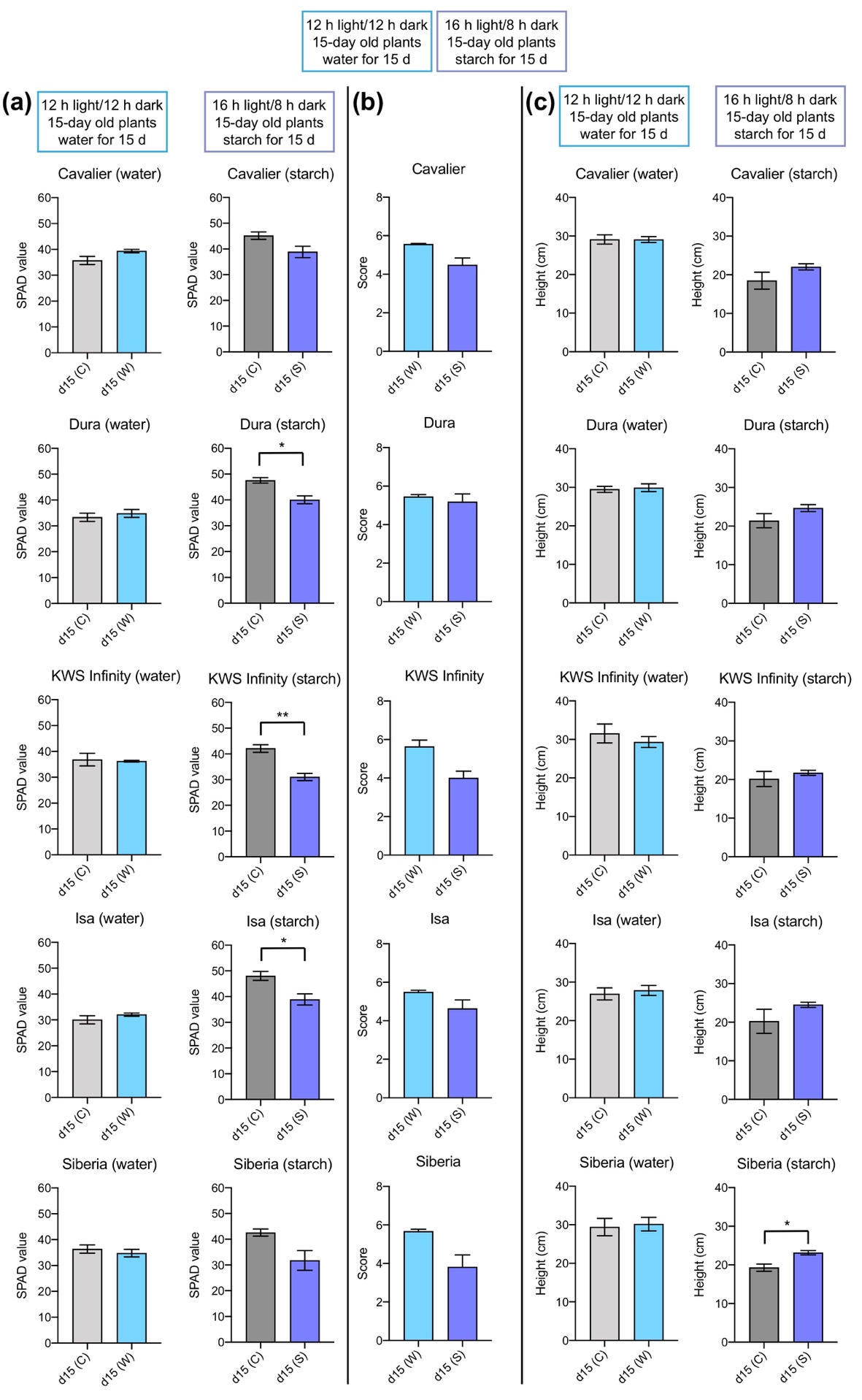


**Figure S3: Effect of photoperiod of the phenotypic differences upon partial submergence.** Fifteen-day old plants were subjected to partial submergence with either a 0.1% starch solution under 16 hours light/8 hours dark at 15°C or water under 12 hours light/12 hours dark at 15°C. All measurements were made on day 15 of partial submergence or control conditions (normal watering). **(a)** SPAD values. Data from 3 independent replicates is shown for treatment with a 0.1% starch solution (3 plants *per* replicate). For plants treated with water, data from 3 (Infinity), 4 (Siberia) or 5 (all other varieties) independent replicates are shown (3 plants *per* replicate, except for one replicate, which had 2 plants due to germination problems). **(b)** Scores. Data from 3 independent replicates is shown for treatment with a 0.1% starch solution (3 plants *per* replicate). For plants treated with water, data from 3 (Infinity) or 4 (all other varieties) independent replicates are shown (3 plants *per* replicate, except for one replicate, which had 2 plants due to germination problems). **(c)** Plant height. Data from 3 independent replicates is shown for treatment with a 0.1% starch solution (3 plants *per* replicate). For plants treated with water, data from 3 (Infinity), 4 (Siberia) or 5 (all other varieties) independent replicates are shown (3 plants *per* replicate, except for one replicate, which had 2 plants due to germination problems). Mean values and standard error of the mean are shown. Statistical significance of the differences between treated and control plants was determined using a Welch’s t-test because of unequal variance and sample sizes. Significant statistical differences are indicated with: ** for p-values $<$ 0.01; * for p-value $<$ 0.05.
